# Alzheimer’s Disease: An Updated Overview of Its Genetics

**DOI:** 10.3390/ijms24043754

**Published:** 2023-02-13

**Authors:** Jesús Andrade-Guerrero, Alberto Santiago-Balmaseda, Paola Jeronimo-Aguilar, Isaac Vargas-Rodríguez, Ana Ruth Cadena-Suárez, Carlos Sánchez-Garibay, Glustein Pozo-Molina, Claudia Fabiola Méndez-Catalá, Maria-del-Carmen Cardenas-Aguayo, Sofía Diaz-Cintra, Mar Pacheco-Herrero, José Luna-Muñoz, Luis O. Soto-Rojas

**Affiliations:** 1 Laboratorio de Patogénesis Molecular, Laboratorio 4, Edificio A4, Carrera Médico Cirujano, Facultad de Estudios Superiores Iztacala, Universidad Nacional Autónoma de México, Tlalnepantla 54090, Edomex, Mexico; 2 Departamento de Neurobiología del Desarrollo y Neurofisiología, Instituto de Neurobiología, Universidad Nacional Autónoma de México, Juriquilla 76230, Querétaro, Mexico; 3 Red MEDICI, Carrera Médico Cirujano, Facultad de Estudios Superiores Iztacala, Universidad Nacional Autónoma de México, Tlalnepantla 54090, Edomex, Mexico; 4 Sección de Estudios de Posgrado e Investigación, Escuela Superior de Medicina, Instituto Politécnico Nacional, Ciudad de México 11340, Mexico; 5 National Dementia BioBank, Ciencias Biológicas, Facultad de Estudios Superiores Cuautitlán, Universidad-Nacional Autónoma de México, Cuatitlan 53150, Edomex, Mexico; 6 Departamento de Neuropatología, Instituto Nacional de Neurología y Neurocirugía Manuel Velasco Suárez, Ciudad de México 14269, Mexico; 7 Laboratorio de Genética y Oncología Molecular, Laboratorio 5, Edificio A4, Carrera Médico Cirujano, Facultad de Estudios Superiores Iztacala, Universidad Nacional Autónoma de México, Tlalnepantla 54090, Edomex, Mexico; 8 División de Investigación y Posgrado, Facultad de Estudios Superiores Iztacala, Universidad Nacional Autónoma de Mexico, Tlalnepantla 54090, Edomex, Mexico; 9 Laboratory of Cellular Reprogramming, Departamento de Fisiología, Facultad de Medicina, Universidad Nacional Autónoma de México, Ciudad de México 04510, Mexico; 10 Neuroscience Research Laboratory, Faculty of Health Sciences, Pontificia Universidad Católica Madre y Maestra, Santiago de los Caballeros 51000, Dominican Republic; 11 National Brain Bank-UNPHU, Universidad Nacional Pedro Henríquez Ureña, Santo Domingo 1423, Dominican Republic

**Keywords:** Alzheimer’s disease, genetics, GWAS, loci, molecular mechanisms, neurodegeneration, neuropathology

## Abstract

Alzheimer’s disease (AD) is the most common neurodegenerative disease in the world. It is classified as familial and sporadic. The dominant familial or autosomal presentation represents 1–5% of the total number of cases. It is categorized as early onset (EOAD; <65 years of age) and presents genetic mutations in *presenilin 1* (*PSEN1*), *presenilin 2* (*PSEN2*), or the *Amyloid precursor protein* (*APP*). Sporadic AD represents 95% of the cases and is categorized as late-onset (LOAD), occurring in patients older than 65 years of age. Several risk factors have been identified in sporadic AD; aging is the main one. Nonetheless, multiple genes have been associated with the different neuropathological events involved in LOAD, such as the pathological processing of Amyloid beta (Aβ) peptide and Tau protein, as well as synaptic and mitochondrial dysfunctions, neurovascular alterations, oxidative stress, and neuroinflammation, among others. Interestingly, using genome-wide association study (GWAS) technology, many polymorphisms associated with LOAD have been identified. This review aims to analyze the new genetic findings that are closely related to the pathophysiology of AD. Likewise, it analyzes the multiple mutations identified to date through GWAS that are associated with a high or low risk of developing this neurodegeneration. Understanding genetic variability will allow for the identification of early biomarkers and opportune therapeutic targets for AD.

## 1. Introduction

Alzheimer’s disease (AD) is a neurodegenerative disease and represents the most common form of dementia (60–80% of all cases of dementia) [1]. At present, an estimated 50 million people worldwide suffer from some form of dementia; however, as a result of the increase in life expectancy rates, it is expected that by 2050, 139 million people worldwide will suffer from some type of dementia [2,3], which will cause major socioeconomic and health system impacts [4].

**Figure 1:**
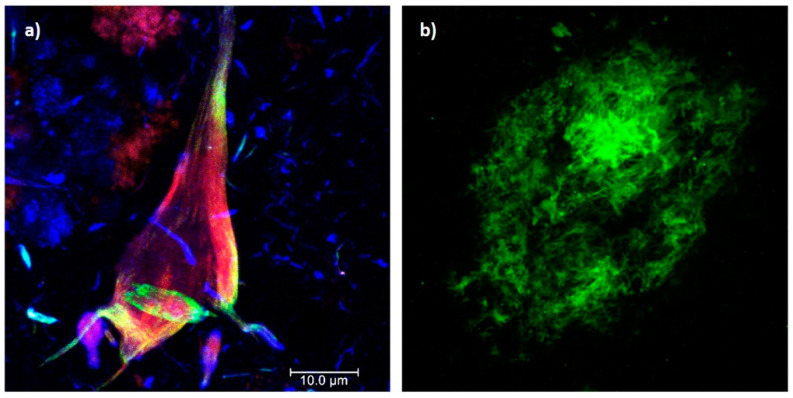
Histopathological hallmarks of Alzheimer’s disease. (**a**) Triple immunofluorescence showing a neurofibrillary tangle (conformational change: green channel; C-terminal tail: red channel; N-terminal of intact tau protein: blue channel). (**b**) Immunofluorescence showing an amyloid plaque (Aβ 1-40: green channel). Photomicrographs at 100X, calibration bar = 10 µm.

AD is characterized by chronic and acquired memory impairment and cognitive deficits in domains, such as language, spatio-temporal orientation, and executive capacity, as well as behavior alterations, all of which lead to progressive loss of personal autonomy [5]. Histopathologically, AD is characterized by two pathognomonic hallmarks (Figure 1) [6]: (1) the intracellular deposition of abnormally phosphorylated Tau protein that promotes the formation of neurofibrillary tangles (NFTs) in the cerebral cortex and subcortical gray matter (Figure 1a); and (2) extracellular aggregates of Amyloid-beta peptide (Aβ) fibrils in the form of neuritic plaques (NPs; Figure 1b). In this context, it has been postulated that endogenous “damage signals”, such as Aβ oligomers, could cause the activation of microglial cells with the consequent release of proinflammatory cytokines, which would trigger signaling cascades in neurons causing hyperphosphorylation and the aggregation of Tau protein. This protein is released when neurons die, triggering microglial cell activation and, therefore, becomes a cyclic pathological process that culminates in neurodegeneration [7,8]. Therefore, both NPs and NFTs are involved in several neuronal processes and ultimately trigger neuronal death [9,10], synaptic alteration, oxidative stress, mitochondrial disturbance, neuroinflammation, alterations in the permeability of the blood–brain barrier (BBB), and neurovascular unit dysfunction [11].

Two forms of AD have been characterized [6]: familial and sporadic. The familial presentation is autosomal dominant, early onset (EOAD) in individuals under 65 years of age (representing 1 to 5% of cases), and characterized by the alteration of specific genes, such as the *presenilin 1* gene (*PSEN1*, 14q24.2), identified in up to 70% of cases with familial AD; the *presenilin 2* gene (*PSEN2*, 1q42.13) and the *Amyloid precursor protein* gene (*APP*, 21q21.3). The sporadic presentation is late-onset (LOAD) and occurs in individuals older than 65 years of age. The main risk factor is considered to be age [1,9,12], but sporadic AD is a complex disorder and other risk factors have been identified, such as the female sex, traumatic brain injury, depression, environmental pollution, physical inactivity, social isolation, low academic level, metabolic syndrome [9,13] and genetic susceptibility, mainly mutations in the ε4 allele of apolipoprotein E (*APOE*, 19q13.32) [1,9], considering a heritability of up to 60–80% [14].

Multiple hypotheses have been formulated to explain the development of AD, including the amyloidogenic cascade, tauopathy, vascular theory, oxidative stress, neuroinflammation, and bacterial infections theory, among others [6,11,15]. However, due to the intricate complexity of the human brain and partial characterization of the polymorphisms associated with AD, the molecular mechanisms involved in each of these hypotheses and their correlation with the genetic load or predisposition of each individual are poorly understood [16]. Therefore, characterizing the genetic risk factors in AD is a priority to understand the several neuropathological events involved.

Recently, genome-wide association studies (GWAS) have allowed the identification of several genes associated with AD; however, the relationship of many loci to the risk of developing AD has not been elucidated [17]. For this reason, this paper aims to analyze the genes that have been associated with a predisposition to developing AD and the possible neuropathological mechanisms involved. This will facilitate the identification of possible early biomarkers and therapeutic targets that can further the development of AD-modifying drugs.

## 2. The Four Classical Genes Associated with Alzheimer’s Disease

AD is a complex and multigenic disease. Familial AD is associated with the genes that encode *PSEN1*, *PSEN2*, and *APP*, which interfere with the physiological processing of the Aβ peptide [18].

More than 300 mutations (221 pathogenic) of the *PSEN1* gene and 80 mutations (19 pathogenic) of the *PSEN2* gene have been identified [19,20]. These mutations have been related to the genesis of familial AD. The spectrum of *PSEN* genes includes missense mutations, small insertions, deletions, and genomic deletions, specifically in *PSEN1* [18]. *PSEN1* mutations cause the most severe forms of AD with complete penetrance, and the onset of the disease can occur as early as 25 years of age. Missense mutations in the *PSEN2* gene may show incomplete penetrance, and carriers show an older age of disease onset than those with *PSEN1* mutations [21]. The missense mutations give rise to altered proteins, which interfere with the fusion of the γ-secretase complex and, secondarily, alter the processing of APP with a consequent increase in the Aβ42/Aβ40 ratio [21].

The *APP* gene encoding this protein consists of a total of 17 exons and encodes several isoforms resulting from the alternative splicing of exons 7 and 8 [22], three of which are relevant to AD (isoforms 695, 751, and 770) and are expressed only in the central nervous system [23]. The last two exons, 16 and 17, encode the portion of APP that, after proteolytic processing, constitutes the Aβ fragment [24]. Interestingly, 73 mutations (32 pathogenic) have been identified [19,20]; the most frequent is the Val717Ile/London mutation. Mutations in the *APP* gene cause an increase in the Aβ42/Aβ40 ratio, as well as an increase in levels of total Tau and phosphorylated Tau in neurons [9,25]. Most patients with trisomy 21 present early pathological changes similar to those of AD [1,26]; however, not all develop the disease. This suggests that the increase in the genetic load of *APP*, which has also been observed in cases of familial AD [27,28], does not cause the development of the disease in all cases.

On the other hand, the main gene that is consistently associated with sporadic AD is *APOE*, which increases the risk for AD three- to eightfold [6,29]:

The physiological isoform, or ApoE-ε3 (Cis112Arg158), is present in 50 to 90% of the healthy population. The main polymorphisms of the *APOE* gene consist of alleles that translate the isoforms of the protein that differ only by a single amino acid substituted at positions 112 and 158: (i) ApoE-ε2 isoform (Cis112Cis158) is associated with the presentation of hyperlipoproteinemia type III; (ii) ApoE-ε4 isoform (Arg112Arg158) has been implicated in atherosclerosis [30,31], and at least one copy of this allele has been identified in 40–65% of patients with AD. However, not all people with copies of this allele develop the disease, representing only a susceptibility risk factor [1]. APOE competitively binds to Aβ receptors (such as the low-density lipoprotein receptor-related protein 1; LRP1) on the surface of astrocytes, thus blocking Aβ uptake, affecting its clearance and promoting the initial seeding of fibrillar Aβ deposition [6,9]. Growing evidence suggests that APOE influences tau-mediated neurodegeneration and microglial responses to AD-related pathologies [30]. For example, one study revealed that the *APOE3ch* variant may have a regionally specific role in modifying the effect of Tau and thus in the severity, progression, and clinical presentation of AD [32]. This suggests that *APOE* polymorphisms would also affect Tau protein processing and strongly predispose one to AD.

## 3. Unraveling the Genetics of Alzheimer’s Disease: What Is New?

**Figure 2:**
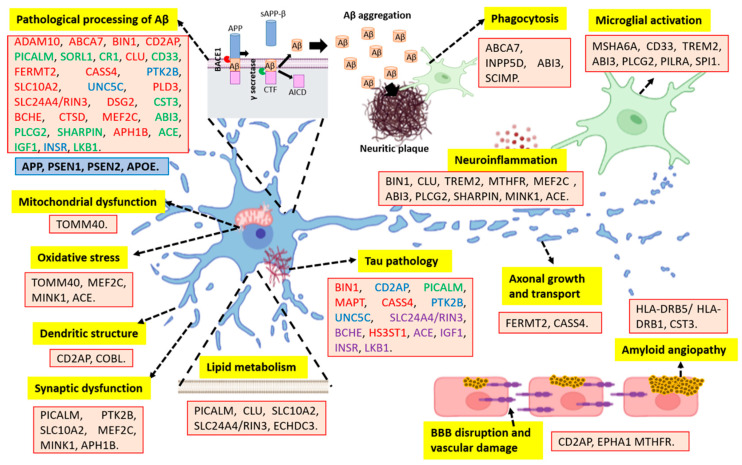
Schematic representation of multiple genes and their association with neuropathological events in AD. The four classical genes associated with familial AD are in the blue box, whereas other novel genes related to sporadic AD are in the red boxes. Word color-coding: red: increased production/aggregation of Aβ or tau; green: alteration in the clearance of Aβ or tau; blue: Aβ- or tau-mediated neuronal damage; purple: Tau phosphorylation. Furthermore, the respective associations with neuropathological mechanisms are indicated in yellow boxes. Abbreviations: AICD: amyloid precursor protein intracellular domain; APP: amyloid precursor protein; BACE1: β secretase; BBB: blood–brain barrier; CTF: C-terminal fragment; sAPP-β: soluble amyloid precursor protein β. This figure was created with biorender.com.

A wide variety of genes in the development of AD have been identified as possible early biomarkers and therapeutic targets. Together, these genes could explain up to 70% of LOAD cases and point to new disease-related phenomena [5], such as the association of these genes with multiple neuropathological events (Figure 2 and Table 1).

According to the amyloid hypothesis, a wide variety of genes identified are involved in pathological Aβ processing (Figure 2 and Table 1), such as the *disintegrin and metalloproteinase domain-containing protein 10* (*ADAM10*), which is the most important α-secretase in the brain that could cause increases in the Aβ42/Aβ40 ratio [33,34,35]. In the same way, the *phosphatidylinositol-binding clathrin assembly protein* (*PICALM*) initiates the polymerization of clathrin, which contributes to NP formation [36,37,38]; *clustered mitochondria protein homolog* (*CLU*), a chaperone that prevents the aggregation of foreign proteins, has been found to inhibit the formation of amyloid fibrils by APP and other genes involved [39,40,41].

However, the pathological Aβ processing is not the only mechanism involved, and it does not explain the complexity of AD. More than 29 risk loci have been identified, as well as more than 215 potential causative genes. These genes are strongly expressed in immune-related tissues and cell types, including the spleen, liver, and microglia cells [42]. In this context, the neuroimmunomodulation hypothesis has been postulated, which proposes that the appearance of AD is a consequence of the response of glial cells to signals of damage that trigger a neuroinflammatory response and the subsequent deregulation of protein kinases and phosphatases that promote Tau protein hyperphosphorylation and oligomerization. Tau oligomers and filaments released after neuronal apoptosis are in turn capable of reactivating microglial cells, thus promoting a deleterious molecular signaling cycle responsible for the neurodegeneration observed in AD and other tauopathies [43,44,45,46]. Supporting this hypothesis, several genes involved in the immune response have been identified (Figure 2 and Table 1). For instance, *transcription factor PU.1* (*SPI1*), involved in the expression of immune-related genes in myeloid cells, could contribute to AD by regulating key pathways associated with the immune response and altering the epigenetic landscape [47,48]. Furthermore, the *myeloid cell surface antigen CD33*, expressed on the surface of microglial cells, is involved in the negative regulation of cytokine production and, therefore, could play a role in AD by modulating microglial activation [49,50]. In the same way, according to the neuroimmunomodulation hypothesis, diverse genes involved in Tau metabolism have also been identified (Figure 2 and Table 1), such as *bridging integrator 1* (*BIN1*), which participates in membrane tubulation through an interaction with microtubule-associated proteins, such as Tau, and other functions, such as endocytosis and intracellular endosome trafficking [29]. *Triggering receptor expressed on myeloid cells 2* (*TREM2*) has been associated with cerebrospinal fluid Tau levels [51]. Likewise, the loss of function of scaffolding *CD2-associated protein* (*CD2AP*), which is involved in intracellular trafficking and cytoskeletal reorganization, has also been related to Tau-induced neurotoxicity, abnormal neurite structure modulation, and BBB disruption [52].

Similar to the immunomodulation hypothesis, there is the infectious one, which could also explain the pathological processing of both Tau and Aβ. The infectious hypothesis proposes that a pathogen (virus, bacterium, prion, etc.) triggers an inflammatory response and promotes the aggregation of Tau and Aβ, which in turn can cause further inflammation [53]. In this context, the *paired immunoglobulin-like type 2 receptor alpha* (*PILRA*), an inhibitory immunoglobulin receptor involved in the regulation of the immune system and a co-receptor of herpes simplex virus type 1 (HSV-1), is related to dysfunctions in microglial activation and may represent a link to infectious factors that have been connected to AD [24,54,55]. Likewise, *APO-ε4* has also been associated with HSV-1 infections [56]. This is extremely important since a close relationship is established between infectious diseases and AD and, therefore, represents an opportunity both for diagnostic and therapeutic strategies.

In addition, the pathological processing of Tau and Aβ, such as other neuropathological events, could be triggered by some genes involved in metabolic disorders (Figure 2 and Table 1); mainly lipid/atherosclerosis pathways and insulin resistance [57]. In this context, the *solute carrier family 10 member 2* (*SLC10A2*) plays an important role in the regulation of cholesterol metabolism, and it has been shown that cholesterol accumulation in neurons leads to neuronal death, memory impairment, and increased Aβ generation [58,59]. Since AD could be considered a metabolic disease mediated in part by insulin resistance [57], it is postulated that the *zinc finger CW-type PWWP domain protein 1 (ZCWPW1*), a protein involved in the positive regulation of the DNA metabolic process, could decrease the risk of LOAD by suppressing insulin resistance [60]. Furthermore, genes involved in the AMP-activated protein kinase (AMPK) pathway have also been associated with the risk of AD by regulation of energy balance and glucose and lipid metabolism [61], autophagy dysfunctions leading to Aβ and Tau pathology [57], and alteration of the synaptic plasticity of hippocampal neurons [62]. Correspondingly, *methylenetetrahydrofolate reductase (MTHFR*), a rate-limiting enzyme in the methyl cycle, can be linked, as high levels of homocysteine have been found in AD patients. Homocysteine is associated with vascular damage, increased inflammation, and endothelial cell dysfunction [63]. Therefore, further studies are required to determine the genetic predisposition between chronic-degenerative diseases (such as diabetes mellitus) and AD, and the neuropathological pathways involved. 

Table 1 summarizes the main genes, their physiological role, and the possible molecular mechanisms involved in the neuropathology of this neurodegenerative disorder, highlighting that it is possible to differentiate genes associated with neuronal homeostasis, including the physiological metabolism of Tau protein and Aβ peptide, calcium, lipids, synaptic plasticity, neuronal proliferation and differentiation, immunoregulation, cytoskeleton stabilization, and mitochondrial complexes, among others. However, in pathological conditions, they are associated with Tau pathology, increased Aβ accumulation, neuroinflammation, mitochondrial and synaptic dysfunctions, oxidative stress, and disruption of BBB, among others. Understanding the physiological and pathological roles of these genes will facilitate the identification of early biomarkers and opportune therapeutic targets. 

**Table 1:** New genes linked to Alzheimer’s disease and their physiological and pathological roles.

Gene/Location	Physiological Role *	Molecular Mechanisms Implicated in AD	Ref.
ADAM10 (15q21.3)	It is the most important α-secretase in the brain and contributes to the non-amyloidogenic pathway of APP metabolism.	Alteration in APP metabolism (through non-amyloidogenic pathway), synaptic plasticity, and hippocampal neurogenesis.	[33,34,35]
ABCA7 (19p13.3)	Modulates lipid metabolism, phagocytosis, apoptosis, and phagocytic of Aβ by microglia.	Alteration in Aβ processing and regulation of APP by β-secretase.	[64,65,66]
BIN1 (2q14.3)	Participates in immune response, calcium homeostasis, apoptosis, endocytosis of synaptic vesicles, and plasma membrane dynamic.	Contributes to Amyloid (through β-secretase activity) and Tau pathology and is related to inflammation, apoptosis, and calcium homeostasis.	[67,68,69]
CD2AP (6p12.3)	Regulates actin cytoskeleton and membrane trafficking through endocytosis and cytokinesis.	Associated with increased Aβ production, Tau neurotoxicity, abnormal modulation of the neurite structure, and altered integrity of the BBB.	[52,70,71]
EPHA1 (7q34)	Plays a role in synaptic development and plasticity, modulating cell migration, angiogenesis, cell proliferation, and apoptosis.	Alterations in immune response and endocytosis, as well as disruption of BBB integrity.	[29,72]
PICALM (11q14.2)	Modulates autophagy, membrane metabolism, internalization of cell receptors, synaptic transmission, removal of apoptotic cells, and endocytic pathways for APP processing.	Dysfunction of Aβ metabolism and APP processing. Possible association with tauopathy, synaptic dysfunction, and altered lipid metabolism.	[36,37,38,73,74]
SORL1 (11q24.1)	Plays a role in endocytosis.	Restricts delivery of precursors to endocytic compartments that promote amyloidogenic clearance.	[75,76]
MS4A6A (11q12.2)	Regulates Ca^2+^ entry and signal transduction of several proteins.	Its variants affect the processing of the TREM2 microglial receptor increasing the risk of AD.	[71,77]
CR1 (1q32.2)	Facilitates the capture and clearance of complement-opsonized pathogens by erythrocytes, monocytes, macrophages, and microglia.	Decreases complement-mediated clearance of Aβ42. Metabolism and clearance of Aβ and its interaction with APOE-4 are related to cognitive impairment and the appearance of LOAD.	[78,79,80]
CLU (8p21.1)	Participates in lipid metabolism, cell proliferation, apoptosis, immune response, and neuronal differentiation.	Alteration in Aβ aggregation, lipid metabolism, regulation of cell cycle and neuronal apoptosis, and neuroinflammation.	[40,41,81]
CD33 (19q13.3)	Involved in the inhibition of immune cell function and cytokine production.	Modulates microglial activation (neuroinflammation) and Aβ clearance through microglial cells.	[49,50,82]
TREM2 (6p21.1)	This microglia receptor regulates proliferation, survival, phagocytosis, and inflammation.	Related to microglial dysfunctions, stress of the endoplasmic reticulum, Tau and amyloid pathology, and neuroinflammation.	[83,84,85]
TOMM40 (19q13.32)	Plays a role in the stabilization of the mitochondrial membrane respiratory chain (Complex I).	Dysfunction of the mitochondrial membrane and subsequent oxidative stress.	[86,87,88]
MAPT (17q21.21)	Related to the assembly and stability of microtubules and neuronal polarity.	Encoding for the Tau protein that is involved in the microtubule disassembly during AD.	[89,90]
FERMT2 (14q22.1)	Participates in cell differentiation, biogenesis, and connection between extracellular matrix adhesion sites and the actin cytoskeleton.	Associated with increasing levels of mature APP at the cell surface, resulting in increased Aβ-peptide production.	[91,92]
CASS4 (20q13.31)	Associated with cell adhesion, cell spreading, calcium signaling, and microtubule stabilization.	Involved in the formation of neuritic plaques and NFT, neuroinflammation, and dysfunction in synapsis, calcium signaling, and microtubule stabilization.	[93,94]
PTK2B (8p21.2)	Involved in the regulation of calcium flux, LTP, neurite growth, synapsis, and angiogenesis.	Contributes to hypoperfusion, vascular permeability, and Tau toxicity.	[93,94,95]
INPP5D (2q37.1)	Participates in the regulation of microglia gene expression.	Involved in microglia with deficient phagocytic capacity, resulting in increased Aβ deposition.	[96,97]
SLC10A2 (13q33.1)	Has an important role in encoding the sodium/bile acid cotransporter, as well as in cholesterol metabolism.	Associated with LOAD by dysfunctional cholesterol metabolism, neuronal death, memory impairment, and increased Aβ generation.	[58,59]
COBL (7p12.1)	Regulates actin cytoskeleton reorganization and neuron morphogenesis and increases the branching of axons and dendrites.	Reduction in the number of dendritic branch points and neurites.	[59]
UNC5C (4q22.3)	Favors apoptosis and directs axon extension and cell migration during neural development.	Contributes to neuronal death, Tau pathology, and Aβ-associated pathways.	[98,99]
PLD3 (19q13.2)	Hydrolysis of membrane phospholipids, which influences the processing of APP.	Promotes the generation of amyloid plaques and cognitive decline.	[100,101]
SLC24A4/RIN3 (14q32.12)	Plays a role in calcium transport and lipid and glucose metabolism.	Associated with Aβ loading and Tau pathology.	[102]
HLA-DRB5/DRB1 (6p21.32)	Encodes proteins for MHC and plays an important role in the immune response, including antigen processing and presentation, and self-recognition by immune cells.	It is associated with the presence of capillary β-amyloid and the development of cerebral Amyloid angiopathy. It also induces microglial activation.	[103,104]
DSG2 (18ql2.1)	Encodes adhesion molecule proteins to promote contact between epithelial cells and other cells.	Mediates APOE-associated Aβ aggregation in neurons.	[105]
MTHFR (1p36.3)	The regulatory connection between the folate and methionine cycles.	Associated with high levels of homocysteine and subsequent vascular damage and neuroinflammation.	[63,106]
CST3 (20p11.21)	Promotes neurogenesis, reduces Aβ deposition, and inhibits fibril formation.	Involved with amyloid angiopathy and cell death induced by oligomeric and fibrillar Aβ.	[107,108,109]
BCHE (3q26.1)	Contributes to acetylcholine inactivation and hydrolysis of neurotoxic organophosphate esters.	Related to NFT and neuritic plaque formation.	[16,110,111]
CTSD (11p15.5)	Favors the activation/degradation of polypeptide hormones and growth factors.	Implicated in the processing of APP/Aβ deposition and autophagy dysfunction.	[112,113]
ZCWPW1 (7q22.1)	Regulation of the DNA metabolic process. Additionally, it is involved in epigenetic modulation.	Suppresses insulin resistance. It may activate the PI3K signaling pathway in neurons.	[60]
MEF2C (5q14.3)	Involved in vascular development, neurogenesis, inflammatory processes, and hippocampal-dependent learning and memory.	Promotes neuroinflammation, cell apoptosis, Aβ aggregates, synaptic plasticity dysfunction, and increases the oxidative stress level.	[114,115,116]
ABI3 (17q21.32)	Regulates actin cytoskeleton organization, cytokinesis, migration, endocytosis, and phagocytosis.	Associated with alterations in microglial migration and phagocytosis, Aβ accumulation, and neuroinflammation.	[117,118]
PLCG2 (16q24.1)	Regulates divergent microglial functions through TREM2 signaling and is involved in the transition to a microglial state.	Correlation with amyloid plaque density, expression levels of microglial marker genes, and neuroinflammation.	[119,120,121,122]
SCIMP (17p13.2)	Regulates MHC-II signaling in B cells and the host’s innate immune responses to danger signals.	Associated with alteration in TLR-mediated microglial phagocytosis via MHC II.	[123]
SHARPIN (8q24.3)	Induces NF-kB activation and regulation of inflammation, and cell death. Furthermore, it regulates angiogenesis and NLRP3 activation.	Associated with neuroinflammation, and defective Aβ clearance that leads to pathogenic Aβ accumulation.	[124,125,126,127,128]
MINK1 (17p13.2)	Controls glutamate receptor signaling, synaptic density, dendrite complexity, actin cytoskeleton reorganization, cell-matrix adhesion, cell–cell adhesion, and cell migration.	It is related to glutamatergic synapse impairment, dysfunction of axon regeneration, neuronal degeneration, neuroinflammation, and increased ROS levels.	[129,130,131]
APH1B (15q22.2)	Serves as a scaffold for the assembly of γ-secretase complex; therefore, it cleaves APP. It also favors excitatory synaptic transmission and plasticity.	Decreases excitatory synapsis and promotes Aβ aggregation and spine formation.	[132,133,134]
HS3ST1 (4p15.33)	Modulates stem cell differentiation and neuronal targeting.	Promotes Tau spreading mediated by Tau binding to the cell surface heparan sulfate.	[135,136]
ECHDC3 (10p14)	Involved in fatty acid biosynthesis in mitochondria and insulin sensitivity.	Alterations in lipid and cholesterol metabolism, such as cognitive functions.	[137,138]
ACE (17q23.3)	Regulates blood pressure, electrolyte homeostasis, synaptic plasticity, and Aβ metabolism.	Associated with cognitive decline, oxidative stress, neuroinflammation, and higher levels of Aβ and Tau load.	[139,140,141]
PILRA (7q22.1)	Involved in immune system regulation and plays a key role in the life cycle of HSV-1.	Correlation between AD and HSV-1. In addition, it is related to a decrease in the inhibition of microglial activation.	[54,55]
SPI1 (11p11.2)	Regulates the immune response and learning-related neuronal activity in the cerebral cortex.	Alters the microglial phenotype and transcriptome, involving interferon pathways.	[47,48]
IGF1 (12q23.2)	Inhibits abnormal Tau phosphorylation and Aβ deposition. Stimulates neurogenesis and prevents apoptosis in the hippocampus.	Associated with Tau and Aβ pathology.	[142,143]
INSR (19p13.2)	The insulin-INSR signaling pathway regulates glucose uptake and release, as well as the synthesis and storage of carbohydrates, lipids, and proteins.	Can activate GSK-3β by initiating the PI3K-AKT signaling pathway and lead to Aβ accumulation and Tau phosphorylation.	[57]
LKB1 (19p13.3)	Plays a role in cell metabolism, cell polarity, apoptosis, and the response to DNA damage by regulating AMPK activity.	Autophagy dysfunction, Aβ accumulation, and Tau phosphorylation.	[57]

Abbreviations: ABCA7: ATP-binding cassette subfamily A member 7; ABI3: ABI gene family member 3; ACE: Angiotensin-converting enzyme; ADAM10: Disintegrin and metalloproteinase domain-containing protein 10; AMPK: AMP-activated protein kinase; APH1B: Gamma-secretase subunit APH-1B; APOE-4: Apolipoprotein E allele 4; APP: Amyloid beta precursor protein; Aβ: Amyloid beta peptide; BBB: Blood–brain barrier; BCHE: Butyrylcholinesterase; BIN1: Bridging integrator 1; Ca2+: Calcium; CASS4: Cas scaffolding protein family member 4; CD2AP: CD2-associated protein; CD33: Myeloid cell surface antigen CD33; CLU: Clustered mitochondria protein homolog; COBL: Protein cordon-bleu; CR1: Complement receptor type 1; CST3: Cystatin-C; CTSD: Cathepsin D; DSG2: Desmoglein-2; ECHDC3: Enoyl-CoA hydratase domain-containing protein 3, mitochondrial; EPHA1: Ephrin type-A receptor 1; FERMT2: Fermitin family homolog 2; GSK-3β: glycogen synthase kinase 3; HLA-DRB5: HLA class II histocompatibility antigen, DR beta 5 chain; HS3ST1: Heparan sulphate glucosamine 3-O-sulfotransferase 1; HSV-1: Herpes simplex virus type 1; IGF1: Insulin-like growth factor 1; INPP5D: Inositol polyphosphate-5-phosphatase D; INSR: Insulin receptor; LKB1: Liver kinase B1 LOAD: Late-onset Alzheimer’s disease; LTP: Long-term potentiation; MAPT: Microtubule-associated protein tau; MEF2C: Myocyte-specific enhancer factor 2C; MHC II: MHC Class II; MHC: Major histocompatibility complex; MINK1: Misshapen-like kinase 1; MS4A6A: Membrane-spanning 4-domains, subfamily A, member 6A; MTHFR: Methylenetetrahydrofolate reductase; NF-kB: Nuclear factor kappa-light-chain enhancer of activated B cells; NFTs: Neurofibrillary tangles; NLRP3: NLR family pyrin domain containing 3; PICALM: Phosphatidylinositol-binding clathrin assembly protein; PI3K: Phosphoinositide 3-kinase; PILRA: Paired immunoglobulin-like type 2 receptor alpha; PLCG2: Phospholipase C gamma 2; PLD3: Phospholipase D family member 3; PTK2B: Protein-tyrosine kinase 2-beta; ROS: Reactive oxygen species; SCIMP: SLP adapter and CSK-interacting membrane protein; SHARPIN: SHANK-associated RH domain interactor; SLC10A2: Solute carrier family 10 member 2; SLC24A4: Solute carrier family 24 (sodium/potassium/calcium exchanger) member 4; SORL1: Sortilin-related receptor 1; SPI1: Transcription factor PU.1; TLR: Toll-like receptor; TOMM40: Translocase of outer mitochondrial membrane 40; TREM2: Triggering receptor expressed on myeloid cells 2; UNC5C: Netrin receptor UNC5C; ZCWPW1: zinc finger CW-type PWWP domain protein 1. * Data source: UniProt.org (accessed on 20 December 2022).

**Figure 3:**
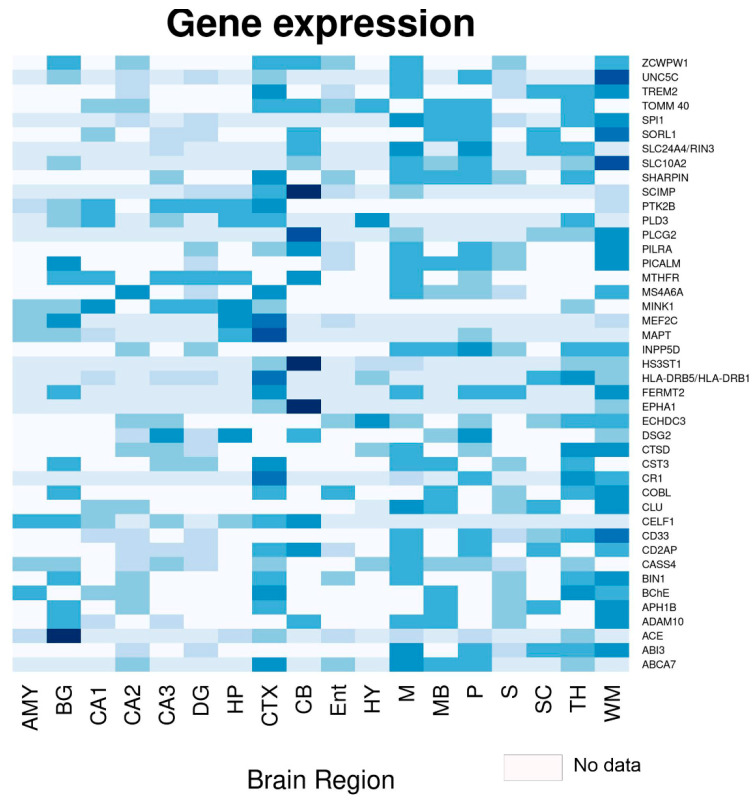
Heat map of the different genes associated with Alzheimer’s disease and their expressions in various regions of the brain and hippocampus. Abbreviations: AMY: Amygdala; BG: basal ganglia; CA1, CA2, CA3: Hippocampal regions; CB: Cerebellum; CTX: Cerebral cortex; DG: Dentate gyrus; Ent: Entorhinal gyrus; HP: Hippocampus; HY: Hypothalamus; M: Medulla oblongata; MB: Midbrain; P: Pons; S: Subiculum; SC: Spinal cord; TH: Thalamus; WM: White matter. Gene expression by brain region was obtained from the human protein atlas (https://www.proteinatlas.org/, accessed on 20 December 2022).

Figure 3 illustrates the expressions of several genes in regions of the brain, highlighting that many of these are highly expressed in the hippocampus, the main neuroanatomical region affected in AD. Remarkably, *CD33, DSG2, MEF2C, MINK1, PTK2B, CD2AP, PICALM, BIN1, MTHFR, PLD3,* and *TOMM40* are highly expressed in the hippocampus. They are related to various pathological processes (Figure 2, Table 1), mainly abnormal Tau and Aβ processing, synaptic dysfunction, and neuroinflammation, highlighting the causal role of the immune system, rather than the immune response as a consequence of the disease. The neuroimmunomodulation hypothesis postulates that AD is a consequence of the response mainly of microglial cells that trigger a neuroinflammatory response and the subsequent neurodegeneration [43,44,45,46]. Furthermore, *CD2AP*, *CD33*, *BIN1*, *CR1*, *PICALM*, *ABCA7*, *TREM2*, and *CLU* are strongly expressed in the hippocampus (Figure 3) and represent highly expressed risk genes in microglia; hence, they are associated with the immune response in AD [144]. This is highly relevant since these genes represent a window for the establishment of early biomarkers and therapeutics focused on limiting the neuroinflammatory environment.

## 4. Impact of GWAS on Understanding Alzheimer’s Disease

From 2005 to the present, multiple genetic studies have tracked most of the genes that conform to the human genome. These genetic studies are known as Genome-wide Association Studies (GWAS) and their objective is to associate certain genes with multiple pathologies or disorders [145,146]. Until 2007, only mutations in the APOE-ε4 allele were reliably associated with increased susceptibility to LOAD. Nonetheless, to date, multiple analyses have been performed with GWAS technology, demonstrating many possible genes associated with LOAD (Table 2). Targeted genetic approaches and next-generation sequencing studies have also identified several low-frequency genes that are associated with a relatively high risk of developing AD, therefore providing insight into the pathogenesis [5]. GWAS have associated more than 40 risk alleles with AD, identifying variants that trigger neurodegeneration, such as lipid metabolism, inflammation, innate immunity, Aβ production and clearance, and endosomal vesicle recycling (as shown in Table 1 and Figure 2) [147,148]. In particular, GWAS have also allowed us to identify those genes related to the development of both EOAD and LOAD [14].

Since genetic variations have been evidenced between different ethnic groups [149], it is essential that researchers perform GWAS in AD across ethnicities and identify polymorphisms associated with each of them. In Table 2, note that most GWAS analyses have been conducted in the Caucasian population and, therefore, are biased by not including other populations and determining the susceptibility of other possible genetic variations involved. The African American population is twice as likely to develop the disease [14]; hence, it is necessary to increase the studies on these populations. In the same way, only one of the studies [150] focused on identifying possible genes involved in AD taking into account the gender of the patients, although women are at a higher risk of developing AD and have worse clinical and pathological outcomes [151]. In addition, some of these studies could be biased, as the sample between controls and patients with AD was not balanced and neither were the risk factors or modifiers of the study subjects, including comorbidities, gender, age range, environmental exposure, and medication, among others.

On the other hand, GWAS have allowed the identification of genetic components that are not related to the neuropathological processes mentioned above (Figure 2), affecting only cognitive reserve, which refers to individual differences in susceptibility to age-related brain changes or AD-related pathology [152]; thus, some people would tolerate more of these changes and still maintain function. In this context, growing evidence suggests that, among cognitively healthy patients with a genetic risk of developing AD, women exhibit better global cognition than men. This event is maintained during the early stages of the disease, despite showing increased Tau pathology, hippocampal atrophy, and metabolic dysfunction [151]. This indicates that although women have greater resilience to the disease, there are still unidentified factors that cause them to have a greater risk of developing the disease.

Although GWAS have been invaluable in identifying candidate genetic variants associated with the disease, the AD risk loci identified to date explain only a small fraction of the heritability of AD [42]; therefore, part of it remains unexplained. One solution would be to increase the sample size of GWAS, as they have already been used to characterize new genetic risk factors in other diseases [14]. Interestingly, GWAS in AD have transitioned from identifying only a couple of novel genes to identifying a large number of previously unreported associations. This is probably due to an increase in the size of the samples and the diversification of the populations studied.

However, the increase in sample size is due to the use of a Russian doll-like design where larger studies include all smaller studies [153], which could limit results when analyzing the same samples multiple times instead of generating new patient data. In support of this, some GWAS [14,42,59,154] have shown that the use of a proxy patient (self-report of the parental history of AD) is useful to favor an increase in the sample size without affecting the validity of the results.

Therefore, future GWAS must adopt a better definition of cases and controls, as well as designs based on the comparison of ethnic groups, sex, and age, since there may be various environmental, exposure, and genetic factors that modify the risk load conferred by each locus. Likewise, these associations should be replicated and validated in multiple populations and followed by a downstream functional dissection to benefit knowledge of pathophysiology [155].

**Table 2:** Genome-wide association studies (GWAS) for Alzheimer’s disease.

Year	Population (Ethnicity/Race)	New Genes Found *	Ref.
2007	British and Americans (*n* = 3870); LOAD = 1808; Ctrl = 2062	APOE	[156]
2008	European ancestry (*n* = 1376).	CD33	[157]
2009	1. French (*n =* 7360); LOAD = 2032; Ctrl = 5238 2. Belgians, Finns, Italians, Spanish (*n =* 7275); LOAD = 3978; Ctrl = 3297	CR1, CLU	[158]
2009	1. British, Germans, and Americans (*n =* 11,789); LOAD = 3941; Ctrl = 7848 2. Europeans (*n* = 4372); LOAD = 2032; Ctrl = 2340.	PICALM	[41]
2010	Spanish (*n* = 2349); LOAD = 1140; Ctrl = 1209	EXOC3L2, BIN1	[159]
2010	Caucasians of German extraction (*n =* 970); LOAD = 491; Ctrl = 479	TOMM40	[160]
2011	1. Europeans (*n =* 9799); LOAD = 4896; Ctrl = 4903 2. Europeans (*n* = 29,544); LOAD = 8286; Ctrl = 21,258	ABCA7, MS4A6, EPHA1, CD2AP	[71]
2011	European Americans (*n* = 3839); LOAD = 1848; Ctrl = 1991	CUGBP2	[161]
2011	1. African Americans (*n* = 1009); LOAD = 513; Ctrl = 496 2. White (*n* = 9773); LOAD = 3568; Ctrl = 6205	PVRL2	[162]
2012	Caribbean Hispanics (*n* = 1093); LOAD = 549; Ctrl = 544	DGKB, GWA-10q23.1 (PCDH21, LRIT1, RGR), HPCAL1	[163]
2012	Polish (*n* = 282); Cases = 141 (94 LOAD, and 47 MCI); Ctrl = 141	GWA-9q21.33	[164]
2012	1. European Americans (*n* = 2440); LOAD = 1440; Ctrl = 1000 2. European Americans (*n =* 6063); LOAD = 2727; Ctrl = 3336	PPP1R3B	[165]
2013	European ancestry and African Americans (*n* = 4689); LOAD = 2493; Ctrl = 2196	PLD3	[166]
2013	1. Icelanders (*n* = 4786); LOAD = 3550; Ctrl = 1236. 2. Americans, Norwegians, Germans, Dutch (*n* = 11,764); LOAD = 2037; Ctrl = 9727	TREM2	[167]
2014	European Americans (*n =* 3656); LOAD = 2151; Ctrl = 1505	APOC1, CAMK1D, FBXL13	[168]
2014	Multiethnic (mostly Caucasians; *n =* 32,346); LOAD = 14,967; Ctrl = 17,379	PLXNA4	[169]
2015	1. Caribbean Hispanics (*n =* 4514); LOAD = 2451; Ctrl = 2063 2. Caribbean Hispanics (n = 5300); LOAD = 3001; Ctrl = 2299	FBXL7, FRMD4A, CELF1, FERMT2, SLC24A4-RIN3.	[170]
2015	Japanese (*n* = 8808); LOAD = 816; Ctrl = 7992	CNTNAP2, GWA-18p11.32, GWA-12q24.23	[171]
2016	European ancestry (*n* = 3467) LOAD = 2488; Ctrl = 979	OSBPL6, PTPRG, PDCL3,	[172]
2017	African Americans (*n =* 5609); LOAD = 1825; Ctrl = 3784	COBL, SLC10A2	[59]
2018	Multiethnic (*n =* 54,162); LOAD = 17,008; Ctrl = 37,154	MLH3, FNBP4, CEACAM19, CLPTM1.	[173]
2018	British. Maternal AD = 27,696; Ctrl = 260,980 Paternal AD= 14,338; Ctrl = 245,941	ADAM10, BCKDK/KAT8 (VKORC1), ACE, TREML2, PLCG2, IL-34.	[154]
2019	European ancestry (*n =* 455,258); LOAD = 71,880; Controls = 383,378	ADAMTS4, INPP5D, HESX1, CLNK, HS3ST1, HLA-DRB1, ZCWPW1 (SPDYE3), CNTNAP2, CLU/PTK2B, ECHDC3, APH1B, SCIMP, AB13, BZRAP1-AS1, SUZ12P1, ALPK2, AC074212.3.	[42]
2019	Caucasian ancestry (*n =* 17,480); LOAD = 2741; Ctrl = 14,739	CEACAM16, BCL3, MIR8085, CBLC, BCAM, PVRL2, APOC1, APOC1P1, APOC4, APOC2, LINC00158, MIR155HG, MIR155, LINC00515, MRPL39, JAM2, C2orf74, ATG10, MS4A6A, ABCB9, ZNF815, TRA2A, MED30, LPXN, IRAK3, N4BP2L2, UQCC, APOBEC3F, SFNa, ESPN, GNAI3, C9orf72, MTMR3	[150]
2019	Non-Hispanic Whites (*n =* 94,437); LOAD = 35,274; Ctrl = 59,163	NYAP1 (SPDYE3), ECHDC3 (USP6NL), IQCK, WWOX.	[174]
2020	African Americans (*n =* 8006); LOAD = 2784; Ctrl = 5222	TRANK1, FABP2, LARP1B, TSRM, ARAP1, STARD10, SPHK1, SERPINB13, EDEM1, ALCAM, GPC6, VRK3, SIPA1L2, WDR70, API5, ACER3, PIK3C2G, ARRDC4, IGF1R, RBFOX1, MSX2, AKAP9.	[175]
2021	British (*n =* 495,000); LOAD = 75,000; Ctrl = 420,000	PILRA, NCK2, SPI1, TSPAN14, SPPL2A, ACE, CCDC6, ADAMTS1, SHARPIN, GRN, SPRED2, ADAMTS4, TMEM163, SIGLEC11, PLCG2, IGHG1, IKZF1, TSPOAP1.	[147]
2022	Japanese (*n =* 3777); LOAD = 1744; Ctrl = 2033	FAM126A, ZFHX4, LGR5, ZFC3H1, OR51G1, OR4X2, ARHGEF4, PRUNE2, MLKL, NCOR2, DMD, NEDD4, PLEC	[176]
2022	European (*n* = 788,989); LOAD = 111,326; Ctrl = 677,663	UNC5CL, EPDR1, WNT3, SORT1, ADAM17, PRKD3, MME, IDUA, RHOH, ANKH, COX7C, TNIP1, RASGEF1C, HS3STS, UMAD1, ICA1, TMEM106B, JAZF1, SEC61G, CTSB, ABCA1, ANK3, BLNK, PLEKHA1, TPCN1, SNX1, DOC2A, MAF, FOXF1, PRDM7, WDR91, MYO15A, KLF16, LILRB2, RBCK1, SLC2A4RG, APP.	[14]

Abbreviations: AD: Alzheimer’s disease; Ctrl: controls; LOAD: Late-onset Alzheimer disease; MCI: Mild cognitive impairment. * Only newly identified genes are shown.

Furthermore, studies that have made associations between genetics and epigenetics through epigenome-wide assays should not be left aside. DNA methylation, an epigenetic modification, plays an important role in regulating gene expression and thus in a wide range of diseases and biological processes [177]. Interestingly, one of these analyses was conducted in a Mexican-American population with mild cognitive impairment (MCI) [62], finding various methylation regions between control and MCI patients, as well as some genes involved in neuronal death, metabolic dysfunction, and inflammatory processes. Highlighting that methylation is transgenerationally heritable and affected by environmental factors [177,178], these processes could explain not only the relationship of genetics with the disease, but also its relationship with environmental and lifestyle factors. For example, exposure to organophosphate pesticides has been shown to promote Tau hyperphosphorylation and microtubule dysfunction [179]. Likewise, it has been shown that the methylation of genes, including *ABCA7*, *BIN1*, *SORL1*, and *SLC24A4* (previously described in Figure 2 and Table 1), were significantly associated with the pathological processing of Tau protein and Aβ peptide. In addition, it was demonstrated that histone acetyltransferase and histone deacetylase inhibitors could increase the level of histone acetylation, and thus have different beneficial effects on AD [180]: (i) improve the expression of genes related to memory; (ii) prevent cognitive degeneration; (iii) decrease the deposition of the Aβ peptide; and (iv) avoid hyperphosphorylation of the Tau protein and the NFT formation. Therefore, the identification of polymorphisms associated with multiple environmental factors through GWAS could favor the development of effective diagnostic and therapeutic strategies.

However, GWAS present certain limitations [181]: (1) they eventually involve the entire genome in disease predisposition; (2) the identification of multiple loci without a clear mechanism can be uninformative and cause confusion with respect to AD; (3) the vast majority of GWAS focus on the European population and lack population stratification; and (4) the scarce data can lead to the fact that the heritability of diseases is not fully explained and therefore fail to detect epistasis in humans, which is the main component of the genetic architecture of complex traits [182].

Despite these limitations, as has been analyzed throughout the manuscript, GWAS have multiple benefits given their diverse clinical applications and allow the identification of new biological mechanisms as well as ethnic variations in the health-disease process. Similarly, GWAS should be promoted in understudied populations, with a different methodological and study design, larger sample sizes, with more clinical data, and extrapolating the associations identified to in silico and preclinical models to take full advantage of the potential this technology offers us.

Notwithstanding the significant advances in our understanding of AD pathobiology through GWAS technology, we have yet to identify a disease-modifying therapy that has demonstrated efficacy in humans. Most of the research conducted over the past 30 years has focused on anti-Aβ therapies [183]; however, all clinical trials with anti-Aβ therapies have been unsuccessful [184]. There are several reasons why these therapies may have failed [16,183]: (i) they are administered too late in the course of the disease when neuronal damage has already become irreversible; (ii) the misfolding and accumulation of the Aβ peptide is not the only cause of neurodegeneration and therefore it is not clear whether the reduction of Aβ levels effectively treats the disease; and (iii) since Aβ is produced by multiple pathways, inhibiting its production is complex. Despite the factors listed above, an IgG1 Aβ monoclonal antibody recently proved effective in phase 3 clinical trials to reduce AD biomarkers and reduce cognitive decline [185] and has already been approved for the treatment of AD by the Federal Drug Administration (FDA) [186].

The multiple genes identified by GWAS support the various theories that have been postulated to explain the development of AD, some of them involved in more than one mechanism, such as *PICALM*, *CLU*, and *CD2AP*, among others (Figure 2, Table 1 and Table 2). This supports the hypothesis that the disease and its treatment should be considered from the multiconvergent theory and not through isolated mechanisms. It has been proposed that our genetic program should protect us from diseases, such as AD by working better under the conditions under which it was designed [187]; however, multiple behavioral and environmental factors could play a greater role in the development of the disease than their consideration by single mechanisms. This becomes even more important due to the aforementioned fact that most therapies against a single mechanism continue to show limited success and, therefore, understanding the disease from a unified theory would improve the results of preventive and therapeutic measures. In this context, multiple pathway-directed therapies may be more effective in slowing or preventing the progression of AD. For example, combined therapy with anti-Tau drugs, such as anti-amyloids, has been suggested, as well as the combination of neuroprotective drugs with anti-inflammatory and anti-oxidative stress effects [188].

On the other hand, it should be noted that genetics is only one factor that can influence the treatment of AD, since other factors, such as age, general state of health, and stage of the disease, can also influence the efficacy of the treatment. Similarly, the use of more general approaches to prevent or delay the onset of AD, such as lifestyle interventions or changes to prevent or delay the onset of AD [12], could be useful: maintaining a healthy diet, engaging in regular physical activity, and participating in cognitively stimulating activities [189,190,191].

In conclusion, this review shows the substantial role that genetics plays in the development of not only EOAD, but also LOAD. The GWAS and other genetic analyses will facilitate the identification of possible biomarkers that allow an early diagnosis of the disease or the risk of developing it in susceptible people, as well as its relationship with other pathological processes. Furthermore, post-GWAS functional experiments can elucidate new targets and avenues for therapeutic intervention. Therefore, the selection of genetically supported drug targets could reduce time and costs and improve the success rate of new drug development.

## Data Availability

Not applicable.
